# Culture of patient care among international nursing students: a focused ethnographic study

**DOI:** 10.1186/s12912-024-01807-1

**Published:** 2024-03-06

**Authors:** Mahboube Shali, Fatemeh Bakhshi, Marzieh Hasanpour

**Affiliations:** 1 Critical Care Nursing Education Department, School of Nursing and Midwifery, Tehran University of Medical Sciences, Tehran, Iran; 2 Research Center for Nursing and Midwifery Care, Non-Communicable Diseases Research Institute, Shahid Sadoughi University of Medical Sciences, Yazd, Iran; 3 Pediatric and Newborn Intensive Care Nursing Education Department, School of Nursing and Midwifery, Tehran University of Medical Sciences, Tohid Squ., Dr. Mirkhani [East Nosrat] St., Tehran, 1419733171 Iran

**Keywords:** Culture, Patient care, International students, Nursing, Focused ethnography

## Abstract

**Background:**

This study was conducted to describe and explain the culture of patient care in international nursing students.

**Methods:**

This qualitative study was conducted using focused ethnography. Participants (*n* = 21) were purposefully selected from non-Iranian international students and their nursing instructors. Data collection included semi-structured interviews, and field-note taking. Data were analyzed with the Roper and Shapira inductive approach of ethnographic content analysis.

**Results:**

The cultural model of the study included the acquisition of cultural competence through acceptance of differences and finding commonalities. Subcategories were: “avoiding cultural bias”, “trying to be on the path of adaptation”, “appealing to the support and companionship of colleagues”, “coping with culture shock”, “acculturation”, “getting help from cultural intelligence”, “cultural empathy”, and “language and communication enhancement”.

**Conclusion:**

Cultural competence is teachable. The pattern of formation is through accepting differences and searching for commonalities. Suggestions for promoting the culture of care among international students include effective use of peer groups and teaching different national ethnicities and cultures.

**Supplementary Information:**

The online version contains supplementary material available at 10.1186/s12912-024-01807-1.

## Background

The ultimate objective of nursing education programs is to develop competent graduates [1]. Clinical instruction is a key component of professional nursing education [2]. Clinical practice instruction is the central component of nursing training [3]. This instruction provides nursing students with professional and communication knowledge and skills [4].

Effective communication between healthcare practitioners and clients is dependent on mutual understanding [5]. Globalization and immigration are the root causes of the cultural diversity of societies. Cultural diversity impairs nurses’ ability to understand patients because of their diverse cultural needs and can impede therapeutic communication. Nurses need to respect different cultures when delivering care and be competent to deliver care with meaningful communication [6]. Cultural competency is the ability to understand different cultures when providing care and includes components such as cultural awareness, cultural knowledge, and cultural competencies [7]. Wesołowska et al., 2018 emphasized the importance of cultural competency in nurses’ perception of time pressure, distress, and sleep difficulties [8].

The importance of preparing healthcare culturally competent graduates has been a focal point for health decision-makers in recent years. Walkowska et al., 2023 declared that improving cultural competence levels among healthcare students is effective in their self-confidence and satisfaction with teaching-learning processes in clinical instruction [9]. While much emphasis has been placed on the important role of cultural competence in ensuring quality care, the issue of cross-cultural nursing continues to be inadequately addressed in education [10]. The growing trend of international students and migrant nurses in many countries has challenged the provision of patient-centered and culturally focused care [11]. Another challenge is teaching nursing students from different ethnic backgrounds about cultural differences so that they can take them into account when delivering patient care [12].

Since 2011, the X School of Nursing and Midwifery has started its activities in the international unit and admitted foreign students from different countries around the world. At the time of the study, 40 international students were studying in different grades of nursing (17 undergraduates, 17 master’s students, and 6 doctoral students). Until the year 2023, 72 students in different levels of nursing (29 undergraduates, 32 master’s, and 11 doctoral) have graduated from this faculty and returned to their home countries. The students were mainly from Ghana, Nigeria, Iraq, Afghanistan, and Ethiopia.

Iran is a country that is ethnically and culturally diversified. However, issues related to cultural care were not adequately addressed in the curriculum of domestic and international nursing students [13]. The challenges concerning culturally-based teaching and learning persist as acknowledged by Mitchell et al., (2017) due to an expanding population of international students enrolled in nursing programs in Australia [14]. Cruz et al., (2017) contended that in Saudi Arabia, although nursing students exhibit cultural competence, the presence of cultural diversity prompts a need for enhancing the cultural learning experience [11]. Additionally, in a study conducted by Palmar et al. (2019), it was found that international nursing students studying in Turkey often encountered an unsettling experience characterized by a fear of patient rejection, leading them to perceive themselves as outsiders within the care environment [15]. However, Sun et al. (2023) reported that China developed a cultural-based educational program for international nursing students during the last decade and helped improve the cross-cultural adaptation of these students [16].

According to the literature, international nursing students experience conflict and confusion as they learn to provide care in an environment different from their own culture and country. Studies have demonstrated that these individuals require targeted support as part of their studies, particularly during their clinical training [14]. Despite it is important to examine the dimensions of this issue, few studies have been conducted, especially in Iran [13]. Studies that indicate effective teaching-learning activities to develop the cultural competence of these students are also fragmented [14].

While engaged in training international nursing students, the researchers observed that despite being subjected to a sequence of cultural trainings, these students encountered various obstacles when practicing in clinical settings. One particular issue that stands out is the inadequate emphasis on comprehensive clinical training as a crucial aspect of nursing education. An additional worry is that these students might have unpleasant experiences with discriminatory and racist conduct due to the cultural disparities leading to confusion. Moreover, Anton-Solanas et al., 2021 highlighted the need to consider students’ perspectives, opinions and experiences when designing cultural nursing education and learning activities [12].

This qualitative study aimed to identify cultural learning experiences of international nursing students in the clinical environment using a focused ethnography. The objective of focused ethnography is to delineate the distinct contextual attributes of a certain group within a given culture, as observed in their ordinary routines. Therefore, this study was designed and conducted to describe and explain the culture of patient care among nursing students of the international unit.

## Methods

### Study design

A focused ethnographic design was used. This nursing research method explores a particular phenomenon as experienced by specific individuals within that context [17, 18]. This method helps to learn and understand about culture. The purpose of this study was to explain the culture of patient care in the clinical environment and related issues among international nursing students in Iran. As a result, we used the focused ethnographic method because this method emphasizes the specific social context and the interactions between people in that context [19]. Focused ethnography enables researchers to tackle specific, detailed, and predetermined questions within a shorter timeframe than what is required by conventional ethnography approach [18].

Considering that focused ethnography focuses on a specific phenomenon, researchers must be familiar with the research area and the context in which the research was conducted [20]. All the authors of the present study had experience working and teaching in the clinical environment as nurses and nursing instructors. The author (MH) had many years of experience teaching international students. The authors could observe and interview students and others who interact with them, such as patients and instructors. The authors (all female) had PhD degree in Nursing and training in the field of qualitative study.

### Ethical considerations

The research received the code of ethics from the Joint Organizational Ethics Committee of the School of Nursing and Midwifery & Rehabilitation at X University of Medical Sciences (Ethical Code: XXX). All participants signed the informed consent form after being informed about the objectives of the study. Maintaining confidentiality of information, and giving participants the right to withdraw from continuing cooperation at any desired time were among the principles observed in the research. The data were anonymized to prevent identification. The authors (MS and FB) collected the data.

### Study setting and participants

The study settings were the international unit of the School of Nursing and Midwifery of X University of Medical Sciences and the departments of the affiliated teaching hospital where the international nursing students spent their clinical courses. Two groups of participants were selected (*n* = 21): international nursing students (*n* = 17) and nursing instructors (*n* = 4). The students were from three levels of education: bachelor’s (*n* = 9), master’s (*n* = 5), and doctorate (*n* = 3). The authors did not contact the participants before the study. Convenience and purposive sampling were used to recruit eligible students and instructors. The inclusion criteria for students included being international students, studying in a nursing school, passing clinical courses for at least two academic terms, experiencing involvement in direct patient care, willingness to participate in the study, and having the ability to express experiences. Instructors had at least one year of teaching experience in a clinical environment with international students.

Each of the participants received an email that was a letter of introduction and an invitation to participate in the study. By replying to the email address included in the invitation letter, they informed the main author of their desire to participate in the study. The author (FB) contacted the potential participants, answered their questions about the study, reviewed the consent form, and discussed the range of activities in the research. The consent form was signed before the first interview. Data collection was continued during three academic terms from February 2019 to August 2021 until data saturation. Saturation in this research meant that no new code was extracted in the process of coding.

### Data collection

The method of data collection in this research was based on the triangulation approach as the basis of ethnographic research [18]. A combination of participant observation methods in the real environment, semi-structured in-depth interviews, field notes on cultural observations and interactions, and memoir writing were used. Observation was the main method of data collection. The researchers concluded their observations by examining the various dimensions of the participants in the ethnography, using the participant observation method. Also, the principles of specific descriptive, focused, and selective observation approaches in ethnographic research were also taken into consideration [17].

A cumulative number of 52 observation sessions (total of 50 h) were recorded. Each observation session lasts 30 to 90 min. During the observations, field notes were written based on the observation guide [21]. Observations were made in hospitals affiliated to the university. Since students often spend clinical training in these hospitals, choosing these hospitals helped the researchers to observe the culture of patient care among international nursing students. The researcher (observer) actively strived to take part in several clinical training sessions. Although the study took place in a Persian-speaking setting, all observations and interviews were carried out in English because students commonly rely on English as their reference language. The researcher remained actively involved in the clinical trainings for an extended period and regularly engaged in friendly discussions with the participants. The researcher actively engaged in this position by closely observing the students activities, including dressing procedures, administering injections, providing equipment to other students, collaborating with the instructor, communicating with patients, administering medication, studying case files, and more. Participation in these activities provided the opportunity for the researcher to closely observe the interactions between the student and the patient during care. The researcher also checked her observations and interpretations of these observations with informed people (nursing students from the international unit) through informal interviews. During the observation, informal interviews were used to clarify the observations. For instance, when the student refrained from engaging in verbal exchanges with the patient, an inquiry was made about the motive behind this conduct, or alternatively, when the student engaged in executing clinical duties such as administrating medication or recording vital signs, he was expected to vocalize his sentiments concerning this provision of care. Also, participant observation helped the researcher in formulating interview questions to better understand the cause of the observed behaviors. To ensure a consistent level of self-awareness regarding bias and minimize its influence during the observations, the researcher employed reflection.

The observations of this study were also classified based on the three methods of descriptive, focused, and selective observation in the ethnographic study. During the descriptive observation, the researcher tried to understand the different parts of the departments, routines, and current activities of the department. In this period, our attempt was made to carefully peruse the instructions, letters from various departments, minutes of internal meetings, conference schedules, posters, and brochures on the walls. The guiding question of this study was: “How do students of the international unit take care of diseases from a different culture?” During this period, the researcher tried to make observations by posing questions such as: To what extent do the appearance and accent of the students in the department command attention? How do the students introduce themselves to the patient? How do patients treat students who have different skin colors? In focused observation, the researcher tried to play the role of participant observer. That is, at the same time as participating in the activities, she also performed the act of observation. Students’ behavior was observed during patient care and interactions with the patient, instructor, colleagues, and nurses. In addition to verbal behaviors, non-verbal behaviors of people were also observed. Informal interviews were employed to provide clarification for the observed behaviors. A few instances of these inquiries are as follows: “Which students like to communicate with patients more verbally? How do students initiate communication? How confident are students in communicating and caring for patients? How much help do students receive from the instructor in patient care?” Selective observation was conducted to answer the questions raised during focused observation. For example, when the client did not allow the international student to take care of him/her, the student’s reaction was questionable for the researcher, so she monitored the student to observe the student’s behavior closely.

Field notes describe the context, unique events, and routines. Also, in the end, it was accompanied by the author’s reflection and memoing. In the reflections, the researcher tried to express her feelings and reactions to the studied social situations without affecting the field notes.

In the present study, when no new data was found, the observations were terminated and the interview process started. Although, after conducting the interviews, some selective observations were needed, which were done. An interview guide was prepared for the interviews [22]. The interview we used in our study was developed for this study. The study interview guide is provided as supplementary file 1. Forty-eight interview sessions were arranged with the participants to clarify and deepen the collected information and confirming the validity of the observations and giving direction to the subsequent observations, 25 of which were informal and not prescheduled with students and instructors. Twenty tree semi-structured in-depth interviews were conducted with students with prior planning. Due to the fact that the sampling coincided with the covid 19 pandemic, six interviews were conducted by phone and the rest were conducted face-to-face. The interviews were conducted by setting a previous time and choosing the interview location or path (for phone interviews) according to the opinion and desires of the participants. At the beginning of the interview, the objectives of the study were explained to the participants, and continued with asking these questions from the students: Express your experience of culture in patient care. In cases where you encountered a patient from a culture different from yours, how did you take care of yourself? Did you face any problems in taking care of them and how did you manage it? The interview questions evolved as the research progressed to gain a better understanding of the culture of patient care among international nursing students. Each interview lasted between 30 and 70 min. Interviews were repeated in two cases with the participants. The interviews were audio recorded with the permission of the participants.

### Data analysis

All collected data (transcripts of interviews and field notes) were analyzed with the inductive approach of ethnographic content analysis [17]. The following five steps were followed during data analysis:

1- The researcher transcribed data from interviews, observations, and field notes, resulting in over 145 pages of text that were analyzed simultaneously with data. The data was managed using MAXQDA software version 12. The researchers (MS and MH) read and coded the transcript data multiple times to understand and attain an overall idea of the content, coding segments for meaning and grouping them into emerging themes, ensuring inter-rater reliability of coding [23].

2- Sorting for patterns: Similar codes were placed in a group or category called primary concepts. Then these concepts themselves were placed in the same class according to similarity. Sub-themes were formed. Main themes were obtained from the combination of sub-themes. The categories were formed from the data and represented distinct symbols with shared relationships or meanings.

3- Outlier identification: Negative cases that did not correspond to the findings of the study were identified and taken into account in the data analysis. The approach enabled the comparison of developing patterns across various data types, such as interviews, field notes, and observations.

4- Generalization with constructs and theories: The categories were refined during data collection to assess data fit, comprehensiveness, and the development of themes. To generalize the findings, the link between the internal meanings (Emic) and the insights of the participants in the study, and the author’s mental interpretations (Etic) of these meanings were found. Then theoretical concepts that include both were made. The findings were compared with existing studies and theories.

5- Note-writing: This step was done simultaneously with data collection and review of interviews, observations, and relevant documents. The memos helped the author to find the relationship between the pieces of data, which led to the emergence of theoretical concepts.

### Trustworthiness

The rigor and trustworthiness of focused ethnography can be evaluated with the criteria of Lincoln and Guba (1985), that is, credibility, dependability, confirmability, and transferability [24]. To increase credibility, the following methods were used: triangulation, immersing in data, prolonged engagement, reflection, and member check. For dependability, a limited literature review was done so as not to cause bias in the process of data collection and interpretation. All interviews were recorded and written verbatim. Two nursing faculties who are proficient in qualitative research were asked to review the research process, data, results, and interpretations. Regarding confirmability, all observations were written in a notebook with the time and date on a daily basis and then entered into the Word file as soon as possible. Moreover, based on the type of observation (descriptive-focused and selective), a separate file was created on the computer. To achieve transferability of the results, the researcher tried to consider maximum diversity in the selection of participants.

## Results

Table 1 shows the demographic information of the participants, including the number in each subgroup, the student’s educational level, age, and gender. Observations provided an in-depth description of the cultural context experienced by international nursing students. The results presented in this section are supported by interview transcripts, field notes, and memos (originally in Persian).

**Table 1:** Demographic information of the research participants

Participants	Gender		Age (mean ± SD)
	Male	Female	
Ph.D. student	3	0	35 ± 1.2
Master students	2	3	28 ± 0.8
Bachelor students	3	6	22 ± 3.2
Faculties	2	2	34 ± 3.4

After analyzing the data, 373 primary codes were extracted, and the information was placed in two main classes and eight sub-classes (Table 2). The cultural model of the study included the acquisition of cultural competence through the acceptance of differences and seeking commonalities. According to the cultural model of the study, nursing students have been trying to acquire cultural competence by accepting differences and searching for commonalities. A student who has achieved cultural competence is a person who has acquired a strong foundation in cultural attitudes, cultural knowledge, and cultural skills. In this case, the student can take care of all kinds of patients with any cultural background. Therefore, these students can assess the cultural needs of clients, design an appropriate plan of care, and provide culturally competent care to them in any situation. In the following, the explanations related to the subclasses of each of the main classes have been discussed.

**Table 2:** Cultural pattern of study, acquiring cultural competence through accepting differences and seeking commonalities

Main themes	Sub-themes	Primary concepts
**Acceptance of differences**	Avoiding cultural bias	Stopping prejudice Facing discrimination Avoiding cultural misunderstandings
	Trying to be on the path of adaptation	Enhancing of self-esteem Dealing with loneliness
	Appealing to the support and companionship of colleagues	Implicit training during care Companionship during care
	Coping with culture shock	Recognizing the tension caused by the awareness of differences
**Finding commonalities**	Acculturation	Maintaining one’s cultural identity while accepting a new cultural identity
	Getting help from cultural intelligence	Adapting with values Cultural awareness Enjoy interacting with a new culture
	Cultural empathy	Improving behavioral skills in dealing with a new culture Putting yourself in the shoes of a patient from a different culture
	Language and communication enhancement	Getting help from body language Preparing a dictionary based on taught terms during the course

### Acceptance of differences

International nursing students are generally different from the patients admitted to the hospital in terms of customs, culture, beliefs, and religious ceremonies. Cultural-based care occurs when students understand the cultural differences of the patient and are sensitive to the cultural difference between themselves and the patient. The process of accepting differences is dynamic and learnable. According to the experiences of the participants, acceptance of differences has been confirmed by “*avoiding cultural bias, trying to be on the path of adaptation, appealing to the support and companionship of colleagues, and coping with culture shock*”.

#### Avoiding cultural bias

Biases led to deviations and mistakes of the mind from correct judgment. The reason can be related to the unconscious part of the mind that has been shaped over the years. Differences in appearance, language, and nationality between nursing students and patients sometimes result in experiencing a sense of discrimination and expose them to prejudice. These prejudices sometimes lead to cultural misunderstandings. In this context, one of the undergraduate nursing students shared her experience as follows:

*“We introduce ourselves and tell the patient that we want to perform a therapeutic procedure for you, they don’t allow us. Because we are different. We cannot force the patient. The patient’s comfort is important to us.” (P6)*.

During the observations of how patients were accepted to receive care by students, we observed how students were introduced and supported by instructors. Patients’ cooperation with the provision of care by students, and the way students were introduced and supported by instructors were also monitored.

*In the cardiology department, sixth semester students are taking care of patients with their instructor. The student is completely different from the patient in terms of skin color. In the first stage, the student acted alone to provide care. The patient does not communicate well, and the patient’s companion does not want to receive care from the student. The student asked her instructor to help her to provide care. First, the instructor greets the patient and introduce the student. The patient asks about the country that the student comes from. The instructor helps the student talk to the patient about her country. This is the beginning of a sense of comfort between the patient and the student. The process of providing care is starting. The patient asks the student what kind of home care is provided for cardiac diseases in her country. The student describes the traditional care of her country for the patient (Field Note 11, October 2020).*

One of the undergraduate students shared her experience as follows:

*“We are not very familiar with Iranian culture. The instructor plays an important role in explaining the clients’ behaviors to us. Behaviors that are not understandable to us. How we can perform actions for the patient and the patient is willing to cooperate with us. As with Other Iranian students cooperate”. (P1)*

#### Trying to be on the path of adaptation

Intercultural adaptation occurs when a person acquires the cultural skills of the host culture. This process takes place through communication and is not possible without communication. Successful interaction and adaptation, in turn, affect students’ contextual performance, which itself is an important factor for improving practice. In providing clinical care, students are making a double effort to gain the patient’s trust and cooperation. Trying to adapt and understand some behavioral differences that are specific to Iranians is one of the issues that have been mentioned. Standing on ceremony (a condition of hesitating to ask for what you want) is one of the cultural characteristics of Iranians that most students have mentioned:

*“*…*gradually, I learned that sometimes Iranians say things that may not be true, but it’s not a lie!… for example, they may want something but are embarrassed and say they don’t want it. Iranians understand situations in which another person is standing on ceremony, but it’s a bit strange for us to make the differentiation between real refusing or hesitation among patients”. (P2)*

In another case, students have pointed out the use of the word apology in Iranians. The adaptation they have had from the Iranian culture is that when performing painful and invasive procedures; In addition to explaining the treatment method, there is also a need to apologize.

*“I want to do something for the patient, for example, change the dressing. The patient is in pain. I must apologize to him… I’m sorry he’s in pain!… it means that you respect the patient” (P8)*.

#### Appealing to the support and companionship of colleagues

In many cases, Iranian students are also members of clinical internship groups of international students. Iranian students and their implicit explanations during the procedures and uncovering the ambiguities for international students have had a great impact on the acquisition of caring culture in students. In the observations made, when the instructor was busy providing care to another student, the students asked for help and companionship from their Iranian teammates to provide care.

*In the internal department, 24 patients are hospitalized in the day shift. Seven students as one internship group entered in the department. The instructor introduces each student to an individual patient. One student is Iranian, and the rest are foreign and cannot speak Farsi. After being introduced to the patient, the students have a lot of difficulty in communicating and gaining the patient’s trust. One of the students looks for the Iranian student. After finding him, he asks for his help in asking questions. On physical examination, they see that the patient has a green cloth tied around his wrist. The Iranian student explains to him that this is a kind of religious belief that makes them more hope for healing. The Iranian student helps other students in the group to understand these signs and beliefs (Field Note 23, January 2020).*

One of the instructors shared her experience as follows:

*“The appearance of the students, their accents, and the way they treat each other is different. It is sometimes difficult for the patients to accept these differences. It is difficult to gain their trust. When one of their group mates, who is Iranian, accompanies them, they can usually do their work more easily”. (p. 11)*

#### Coping with culture shock

Being in a new cultural context and the tension caused by facing cultural differences has created a sense of culture shock for students. In this case, students experience a lot of anxiety and worry and have a sense of uncertainty in providing care to the patient. One example of culture shock is when they face differences in roles, expectations, values, and feelings with their identity. In some cases, culture shock has been experienced due to the inability to understand native traditions and customs, social interactions, and native language.

In this context, one of the instructors shared his experience as follows:

*“We see this shock when students go through their first clinical course and they still don’t have enough cultural knowledge. Of course, taking care of an Asian Iranian patient is different from taking care of an African patient. The nature of care is the same… however, culture is an effective factor in patient acceptance of care… in this case, the patient’s culture is different from the care provider’s. Sometimes the student cannot accept it and it takes time to introduce the new culture to the student with explanations and support. (p. 13)*

### Finding commonalities

Although there were many cultural differences between the participants and the patients, the students found some cultural commonalities with the patients. To achieve the goal of finding commonalities, the students have used “acculturation”, “getting help from cultural intelligence”, *“cultural empathy” and “strengthening language and communication”*. Each subcategory with supporting quotes from the participants is explained below.

#### Acculturation

The participants in the research, looked for commonalities while providing care to the patient. In many cases, they acquired some aspects of the new culture and at the same time, they kept their native culture. Accepting the new culture in students helped them in providing care to patients. One of the students shared her experience in this field as follows:

*“For example, we have to make eye contact when talking. In our culture, this is a very bad thing. We don’t have direct eye contact with the audience. In Iran, you must have proper eye contact with the patient to communicate. Too much eye contact, to look less, or to break eye contact, each has a meaning for a person that we should be familiar with”. (P3)*

#### Getting help from cultural intelligence

Many students need help from their cultural intelligence to find commonalities, adapt to the culture and provide care to the patient. Adapting to different values, traditions, and customs, and working in a different cultural environment requires cultural intelligence. Cultural intelligence is the intellectual and practical understanding of the external and internal aspects of people. This intelligence helps to improve the ability to understand differences and enhance human interactions and prevents conflicts caused by cultural differences. Taking help from cultural intelligence gives students the ability to understand the behavioral patterns of patients and as a result, reduce interpersonal communication barriers. In this context, one of the students shared her experience as follows:

*“We may have different values and beliefs. Previously, I experienced caring for patients from different cultures in my country. I enjoy adapting to a new culture and I know that to provide holistic care I must consider cultural differences.” (P6)*.

Concerning the ability to get help from cultural intelligence in providing patient care, one of the instructors who have a long history of working with international students, shared his experience as follows:

*“All students are not the same. I had several internships in different departments. Some students are interested in getting to know the patient’s culture and they ask and are enthusiastic and even make the patient happy… these students are more successful and practice better”. (p. 13)*

### Cultural empathy

The ability to understand and communicate the feelings and thoughts of a patient with a different culture requires empathy. In this case, students can understand and share the feelings of patients in the situation if they experienced it themselves. Each of the students has adopted a special strategy to empathize with patients. Finally, in a summary of all strategies, cultural empathy according to the participants’ experiences consisted of the followings: a kind of response, deep and mutual sense, and understanding, resulting from empathic value and expectation, which often includes cultural exchange.

*“I know that sometimes I need to put myself in the shoes of the patient to understand his/her needs… to address what the patient expects from me. Sometimes my perception is different from the patient’s. Because my culture and situation are different from him/her. I must be able to consider the patient’s needs from the perspective of the patient. (P5)*

### Language and communication enhancement

Language proficiency refers to a person’s ability to understand, speak, read, and write in a language. According to the participants, one of the most challenging parts of adapting to the culture is communicating both verbally and non-verbally. Practicing based on the patient’s expression and non-verbal cues affects the therapeutic relationship. Another challenge is the presence of cultural variation in Iran. In this case, students have also mentioned encountering Iranian patients who speak with different accents and dialects such as Turkish, Northern, Kurdish, and Arabic. One of the Ph.D. nursing students shared his experience as follows:

*“We understand the Persian language. But sometimes many Iranians speak Persian with a heavy accent. The pronunciation of words is different, and I cannot understand. In these cases, I take help from the patient’s family who speaks Farsi”. (p. 14)*

Regarding language enhancement, one of the master’s students shared her experience as follows:

*“We tried to improve our Farsi language… yes… I have many friends in the faculty and they helped me, besides, we also helped each other. If the patient says something that we don’t understand, we ask each other and note the new word”. (p. 15)*

## Discussion

The present study was conducted to describe and explain the culture of patient care among nursing students of the international unit. According to the findings, the cultural pattern formed in the participants includes the acquisition of cultural competence through the acceptance of differences and finding commonalities.

One of the subcategories of research includes avoiding cultural bias. Misperceptions and feelings of discrimination were among the things that the participants had experienced. Considering the cultural difference between the international students and the patients, cultural skills help to establish proper communication, adapt the correct physical distance, and avoid cultural misunderstandings [25]. Cultural bias causes inequalities, discrimination, misunderstandings, and stereotyped behaviors in providing care. Whereas, culture-oriented care reduces treatment inequalities, and patients are treated according to their cultural needs [26].

In the next emerged category, students were constantly trying to adapt to the new culture. A challenge that was bittersweet for the students. According to Bandura’s social cognition theory, when an event is perceived as a challenge for a person, the person makes more effort to solve and adapt to that challenge. Perseverance in this effort results in self-efficacy. Students have reached this level of mastery and self-efficacy by trying to master the challenge of cultural differences. This experience cannot be replaced by other experiences and can only be obtained by studying in a foreign country. This experience may not be created by simulating, playing movies, or lecturing people [27].

Another category is dealing with culture shock. The inability of people facing a new culture to avoid cultural shock is created through verbal and non-verbal actions when interacting with people from a different culture [28]. Many factors can affect culture shock. Previous research shows that people’s personality traits, demographic factors, and organizational support are the main factors that affect culture shock [29, 30]. Some personality traits, such as cultural flexibility, racism, nervous reactions, communication, and interpersonal skills, and the desire to communicate, have a greater impact on culture shock [31].

In the direction of acculturation and adaptation to differences and commonalities, many students have sought help from cultural intelligence. People with higher cultural intelligence are more easily accepted by groups with different cultures; Because these people can establish a good relationship with people of different cultures, and they get social support from different networks. They also use these capabilities to reduce and resolve their culture shock [32]. Improving cultural intelligence in students is a challenge and is important for successful care quality improvement [33].

Strengthening language and communication is another subclass that is mentioned in the present study. Establishing communication is often the most important problem in working with clients who have different cultural backgrounds, and to establish a relationship between the patient and the nurse and promote better interactions, it is necessary to create effective communication [17]. In the present study, the students in many cases have taken help from their peers who speak the same language [34]. In their study, Butow, 2015 also pointed out that another strategy used by nurses is to use a nurse who speaks the same language as the patient to take care of such patients [35].

According to the findings of the present study, to provide complete care to patients by international students, awareness of the culture and the ability to adapt to the culture requires individual and acquired capabilities. Training to improve cultural skills is a good suggestion to improve these capabilities. Young and Guo (2020) highlighted in their study that the advancement of education and cultural competence among nurses continues to face numerous challenges and barriers [36]. Harkess & Kaddoura, 2016 also states that adding cultural competency content to the curriculum is a challenge for nursing schools [37]. In Heydari et al., 2015 highlighted that most of the participants believed that curriculum planning in Iran lacks consideration of how to take care of patients with different cultures [38]. Mousavi & Karimi, 2014 stated in a review study that cultural competence is an important category in the education of medical sciences today and in different parts of the world [39]. Cultural competence education and its measurement in the medical sciences departments is recently considered [40].

This research also had limitations. We conducted interviews in English. In many cases, the students did not have sufficient English proficiency. We asked from students of their language to better understand the concepts. Iran has a high cultural diversity. However, there are many similarities between Iranian culture and other Asian ethnicities, which helps to improve the generalizability of the study results.

## Conclusion

Being on the path of adapting to the new culture is somewhat dependent on the cultural context and cultural intelligence of the students. However, cultural competence is also teachable. The pattern of cultural competence formation is achieved through accepting differences and searching for commonalities. Considering this pattern in the curriculum planning of international nursing students can help them in forming a culture of care. A revision on clinical educational planning can be created by teaching different ethnicities and cultures. Also, the use of movies and animations that better portray different cultures can be effective while integrating with novel teaching methods. Improving the ability of faculty members in teaching culture and effective use of peer groups are other suggestions for promoting the culture of care among international students. Cultural modeling in these students facilitates the management of clinical stress in them and the promotion of other professional nursing competencies. The research outcomes suggest the need for further studies aimed at enhancing cultural intelligence and communication proficiency in international nursing students. Also, research on the impact of culture-based curriculum (especially for cultures with high complexity and tradition) should be conducted in various geographical regions.

### Electronic supplementary material

Below is the link to the electronic supplementary material.

Supplementary Material 1

Supplementary Material 2

## Data Availability

No data are available. The collected data in this study are confidential interview transcripts that are not available for sharing, but may be available from the corresponding author on reasonable request.
