# Supplementary material for: Culture of patient care among international nursing students: a focused ethnographic study
Source: BMC Nurs. 2024 Mar 6;23:163. doi: 10.1186/s12912-024-01807-1 (PMC10916181; doi:10.1186/s12912-024-01807-1)
Supplement: Supplementary file 1 — Supplementary Material 1 [file 12912_2024_1807_MOESM1_ESM.docx]

**Study Information**

This is an interview guide for research entitled “Culture of Patient Care Among International Nursing Students: A Focused Ethnographic Study” with grant number “99-02-100-46849”.

International nursing students experience conflict and confusion as they learn to provide care in an environment different from their culture and country. Although it is important to examine the dimensions of this issue, few studies have been conducted, especially in Iran. Studies that indicate effective teaching-learning activities to develop the cultural competence of these students are also fragmented. This qualitative study aimed to identify the cultural learning experiences of international nursing students in the clinical environment. Therefore, this study was designed and conducted to describe and explain the culture of patient care among nursing students of the international unit with a qualitative approach and focused ethnography.

**Invitation request:** We invite you to help us achieve the research goals by answering the following research questions after completing the consent form.

**Research team information:**

The principal investigator of this research is Dr. Marzieh Hasanpour, PhD and Professor of Nursing, NIDCAP Professional, School of Nursing and Midwifery, Tehran University of Medical Sciences, Tehran, Iran.

Dr. Mahboobe Shali and Dr. Fatemeh Bakhshi will conduct the interviews.

Contact information: Postal Address: Tohid Squ., Mirkhani [East Nosrat] St., Postal Code: 1419733171, Phone No.: +98(0)21-61054413 Fax: +98(21)66904252. Email address:

[m-hasanpour@sina.tums.ac.ir](mailto:m-hasanpour@sina.tums.ac.ir)

As we have mentioned in the consent form, all information collected during the study will be kept safe and secret. Moreover, we will not use your name or any other identifying information; everything you say will only be used for research purposes. Each interview session will last about 30-60 mins.

**Main interview questions:**

The main questions that we are going to ask you are:

- What do you think about the culture in patient care?
- What is the importance of paying attention to patients' culture in care?
- How do cultural differences change your patient care?
- How do you ensure that your patient's culture is respected?
- What are the required competencies for nursing students to provide culturally respected care to the patient?
- How do you care about your patient culture?

We also will be going to ask probing questions (based on what goes on in the interview) as follows for deeper understanding:

- Tell me more
- Why do you feel that way?
- Why was that important to you?
- How did that affect you?
- Can you explain what you mean by that?
- What was your contribution?
- What do you think was making it work?
- How has it changed you?

**We appreciate your contribution in advance.**
